# Predictors of Post-ERCP Pancreatitis (P.E.P.) in Choledochal Lithiasis Extraction

**DOI:** 10.3390/jpm13091356

**Published:** 2023-09-05

**Authors:** Adrian Boicean, Victoria Birlutiu, Cristian Ichim, Samuel B. Todor, Adrian Hasegan, Ciprian Bacila, Adelaida Solomon, Adrian Cristian, Horatiu Dura

**Affiliations:** 1 County Clinical Emergency Hospital of Sibiu, 550245 Sibiu, Romaniavictoria.birlutiu@ulbsibiu.ro (V.B.); adrian.hasegan@ulbsibiu.ro (A.H.); ciprian.bacila@ulbsibiu.ro (C.B.); solomonadelaida@gmail.com (A.S.); adrian.cristian@ulbsibiu.ro (A.C.); horatiu.dura@ulbsibiu.ro (H.D.); 2 Faculty of Medicine, Lucian Blaga University of Sibiu, 550169 Sibiu, Romania

**Keywords:** Post-ERCP pancreatitis, P.E.P, ERCP, ratio dynamics, predictive markers

## Abstract

In the present era, post-ERCP pancreatitis (PEP) stands out as one of the most commonly occurring complications associated with endoscopic choledochal lithiasis extraction. The ability to predict the occurrence of such an event, particularly by utilizing absolute values and ratio dynamics of the emergency blood tests, constitutes the primary step in effectively managing a patient with a complex pathology. The study involved 134 patients who performed ERCP to extract choledochal lithiasis (*n* = 48 with PEP and *n* = 86 without PEP). The results revealed increased risks of post-ERCP pancreatitis in women and lower risks in those who benefited from manipulation of the main bile duct with the Dormia probe and dilatation balloon (OR: 2.893 CI 95%: 1.371–6.105, *p* = 0.005 and respectively OR: 0.346 CI 95%: 0.156–0.765, *p* = 0.009), without biliary stent placement. Moreover, the results brought novel elements to the literature, showing that higher values of CRPR (OR: 4.337 CI 95%: 1.945–9.668; *p* < 0.001), TBIR (4.004 CI 95%: 1.664–9.634; *p* = 0.002) and NLR post-ERCP (3.281 CI 95%: 1.490–7.221; *p* = 0.003) are predictive for PEP. Nevertheless, lower total bilirubin levels upon admission are predictive of PEP with an OR of 5.262 (95% confidence interval: 2.111–13.113, *p* < 0.001).

## 1. Introduction

Endoscopic retrograde cholangiopancreatography (ERCP)is a complex intervention used to investigate and treat some pancreatic-biliary disorders [1]. This procedure has a variety of indications, most commonly for extracting choledocholithiasis and unblocking the main bile duct [2]. This technique was revolutionary in medicine, but it also entails multiple risks that must be considered. Post-ERCP pancreatic reactions are particularly noteworthy because they occur frequently in medical practice and can lead to major complications [3]. Although ERCP is not a relatively new technique, many unanswered questions and perhaps just as many unknowns regarding complications exist.

The instruments used in retrograde endoscopic cholangiopancreatography can have a significant impact on post-interventional complications and patients’ clinical and paraclinical conditions. Additionally, an important study found that the risk factors linked to the underlying pathology should be confirmed by the risk factors linked to the procedure [4].

In recent years, blood tests have been used to predict pancreatic reactions after ERCP, but extensive studies have remained limited to amylase and lipase [5,6,7]. It has been found that some blood tests, which are not routinely collected, may also play a role in the occurrence of PEP-post-Endoscopic retrograde cholangiopancreatography pancreatitis [8,9].

The objective of our single-center retrospective study was to identify variables and to examine the dynamics associated with a higher risk of post-ERCP pancreatitis (PEP). By analyzing retrospective data from our center, we aimed to gain insights into the factors contributing to PEP development. By exploring the dynamics associated with PEP, we aimed to uncover patterns and trends that could provide valuable insights into its prevention and management. Understanding the interplay between various factors, such as procedural techniques and patient characteristics, can help healthcare professionals make informed decisions and implement strategies to mitigate the risk of PEP.

## 2. Materials and Methods

**Figure 1:**
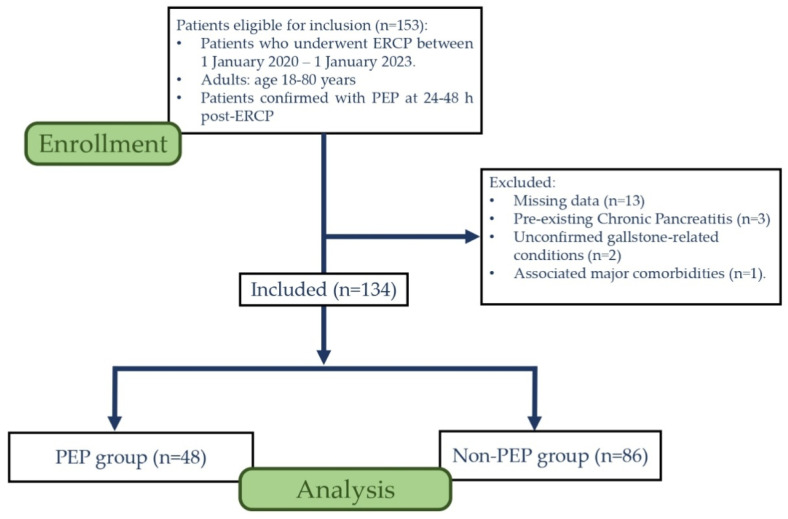
Study flowchart. Abb.-ERCP-Endoscopic Retrograde Cholangiopancreatography; PEP–Post-ERCP Pancreatitis.

Our research involved a retrospective, single-center observational study in the County Clinical Emergency Hospital of Sibiu. We focused on patients who underwent ERCP during their hospitalization and developed pancreatitis. We selected 134 patients hospitalized between 1 January 2020 and 1 January 2023, based on specific criteria: patients aged between 18–80 years who underwent ERCP and developed PEP at 24–48 h post-interventional. We excluded patients with missing data and pre-existing Chronic Pancreatitis, Unconfirmed Gallstones-associated conditions, and patients with major comorbidities (Figure 1). The main aim of the study was to appraise the occurrence of PEP. Pancreatitis should not be confused with a simple rise in amylase levels post-intervention (an aspect that occurs in many cases) [10,11]. In this regard, diagnosis of PEP was established as a threefold increase in serum amylase levels accompanied by clinical symptoms such as new-onset abdominal pain, according to the consensus definition [12], while Elevated Pancreatic Enzymes (EPE) are defined as an increase in amylase levels between 100–300 U/L [10,11].

Since the study was conducted retrospectively, the requirement for informed consent from the patients was waived. We collected laboratory data and clinical records using the hospital data archive. A team of experienced clinicians analyzed all the data and diagnostic information. Comorbidities, demographic characteristics, procedures, and the key outcome (PEP) were extracted from the electronic database. Laboratory assessments included full blood count, coagulation markers, biochemistry, inflammation markers and other relevant indicators. Data analysis was performed in two steps. Categorical variables were shown as numbers (percentages %) and compared using the Chi-square test. For continuous variables, we assessed the normality using the Shapiro-Wilk test. As most of our data were skewed, we utilized Spearman’s correlation coefficient (rs). Continuous variables were reported as median [interquartile range (IQR)] and compared using the Mann-Whitney U test. The Kruskal-Wallis test was performed to assess differences in variables with more than two categories. Optimal cut-off values were determined using receiver operating characteristic (ROC) curves and the Youden index (sensitivity + specificity − 1). To identify predictive factors for PEP, we employed univariable and multivariable logistic regression analyses. Odds ratios (OR) and 95% confidence intervals (CI) were reported for the analysis. Linear regression was used to determine predictive indicators for continuous variables. The goodness of fit model analysis was performed to establish hierarchies between statistical models containing absolute values and ratios by calculating the AUC of the predicted probabilities for each multivariable model. A *p*-value under 0.05 was considered statistically significant. IBM SPSS version 22 was used for the statistical investigation.

## 3. Results

The present study involved 134 patients in the final analysis. Criteria for inclusion were mentioned before. Fundamental patient characteristics observed in those who underwent ERCP are presented in Table 1. There was no significant age difference between non-PEP and PEP groups. However, we can observe a higher incidence of female gender in the PEP group (Table 1). All patients received intrarectal indomethacin before intervention and underwent endoscopic sphincterotomy to minimize the intervention time. No patient went through pancreatic stent placement. As expected, in the PEP group, the levels of lipase and amylase after ERCP were significantly higher, together with neutrophil count and Neutrophil-Lymphocyte ratio (NLR). Total Bilirubin (TB) levels on admission were lower in the PEP group (Table 2).

Preliminary correlation analysis of the parameters showed little to almost no correlation with PEP, except for the value of Total bilirubin (TB) on admission which was negatively correlated with PEP incidence (rs = −0.256, *p* = 0.003), Conjugated Bilirubin post-ERCP (rs = −0.328, *p* = 0.006), and NLR (neutrophil-lymphocyte ratio) post-ERCP who was positively correlated with PEP (rs = 0.242, *p* = 0.006). Additionally, we conducted the dynamic of markers and their association with PEP status using ratios. We defined normal ratios for each parameter by dividing the value after ERCP with the value on admission and inverted ratios for variables negatively correlated with PEP. We ran the correlation analysis again, and we showed that C-reactive protein ratio (CRPR), Neutrophil count ratio (NCR) and TBIR (Total bilirubin inverted ratio) are correlated with PEP (rs = 0.294; *p* = 0.001, rs = 0.262; *p* = 0.003 and rs = 0.205; *p* = 0.018). To establish cut-off points, we executed the ROC curves for all correlated variables (we excluded Neutrophil count ratio and Conjugated bilirubin from the analysis to avoid multicollinearity since both variables are correlated with NLR and Total bilirubin). Areas under the curve and CI 95% were presented (Table 3). Optimal cut-off points for CRPR, TB inverted ratio, NLR after ERCP and TB on admission were: 1.1 (sensitivity 67%, specificity 61.9%), 0.75 (sensitivity 79.2% and specificity 47.8%), 3.7 (sensitivity 65.2% and specificity 61.9%) and respectively 2.65 mg/dL (sensitivity 83.3% and specificity 48.8%). Then, we defined continuous variables as dichotomic variables related to the cut-off points.

We assessed the predictive role of the proposed variables by conducting a binary logistic regression in univariable and multivariable analysis. Univariable and multivariable analysis demonstrated that higher CRPR, TBIR and NLR post-ERCP values predict PEP (Table 4). Nevertheless, lower total bilirubin (TB) levels upon admission are predictive of post-endoscopic retrograde cholangiopancreatography pancreatitis with an odds ratio of. Moreover, female gender was predictive for PEP and combined Dormia basket and ERCP balloon dilation, which predicted the absence of PEP (Table 4). In multivariate analysis, adjustments were made for sex, age and comorbidities like Hypertension and Diabetes.

**Figure 2:**
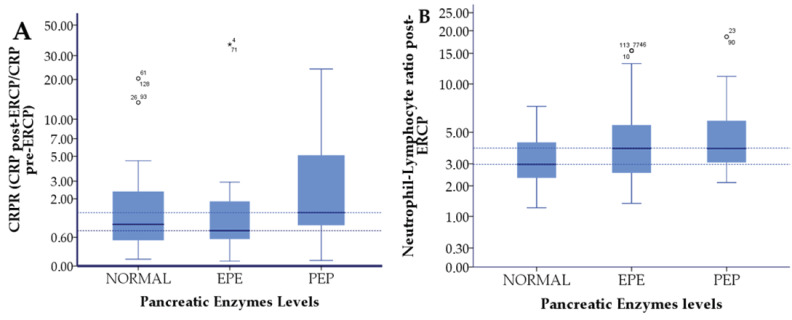
Kruskal-Wallis test shows significant differences in patients with Normal, Elevated pancreatic Enzymes (EPE) and Post-ERCP Pancreatitis (PEP) for (**A**): C-Reactive Protein Ratio and (**B**): Neutrophil-Lymphocyte Ratio. * Dotted lines flag pairwise statistical significance (*p* < 0.05).

Additionally, we divided patients into three groups based on the amylase levels after ERCP from 0–100 U/L-normal levels (NL), from 100–300 U/L Elevated pancreatic enzymes (EPE) and >300 U/L Post-ERCP pancreatitis (PEP) and assessed differences of the predictive markers on this subgroup. Kruskal-Wallis test showed differences in these subgroups for CRPR, NLR post-ERCP and DB levels post-ERCP (Table 5 & Figure 2). Furthermore, pairwise analysis showed that significant differences are registered as follows: for CRPR- between the EPE group and PEP group (*p* = 0.012) and for NLR post-ERCP—between the Normal group and PEP group (*p* = 0.012) (Table 5) (Figure 2). Moreover, multilinear regression showed that CRPR is the only variable correlated with amylase levels post-ERCP (B: 20.5 CI 95%- 0.7–40.3; *p* = 0.043).

**Figure 3:**
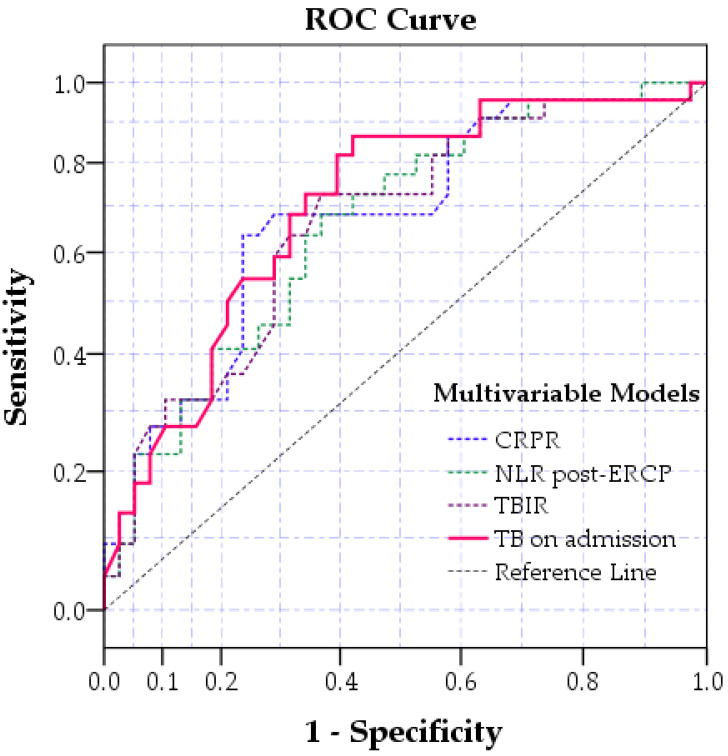
The goodness-of-fit model analysis of each multivariable model included shows the highest AUC for Total Bilirubin (TB) on admission (continuous pink line) compared to the other models. Abb.-CRPR-C-reactive protein ratio; TBIR-Total bilirubin inverted ratio; NLR-neutrophil-lymphocyte ratio; ERCP-Endoscopic retrograde cholangiopancreatography.

The goodness of fit model analysis between multivariate models containing absolute and ratio variables showed that TB levels on admission (AUC = 0.731) are the most precise in assessing outcome prediction compared to NLR-post-ERCP, TBIR and CRPR (Figure 3 and Table 6).

## 4. Discussion

The results of our study indicate that PEP occurred in 48 (35.8%) patients, with no mortality registered in the selected period. No age difference was observed among patients with or without PEP (Table 1). Female gender was more common in the PEP group (47.1% vs. 24.2%; *p* = 0.006) (Table 1). According to epidemiological data, post-endoscopic retrograde cholangiopancreatography pancreatitis (PEP) occurrence rate ranges from 1% to 10%. However, there have been instances where the incidence has been reported as high as 30% [13]. Moreover, when multiple risk factors are present, such as female gender, Sphincter of Oddi dysfunction, laborious interventions or normal bilirubin levels, the risk of PEP can increase even further, up to 40% [14].

### 4.1. Clinical and Interventional Factors

#### 4.1.1. Gender Disparities Regarding PEP Risk

Many research papers focused on sex—specific risk of PEP. However complex the literature analysis is, it remains clear that the female sex is one of the elements that accurately predicts PEP, even in the elderly [3,15,16]. According to this material analysis, the same result was obtained, but there is no consensus regarding the pathophysiological mechanism. It is unlikely that hormonal factors are implicated in this risk factor since it is preserved in the elderly. At this time, there is no concrete evidence of anatomical gender variations of the pancreatic pathway that might predispose females to PEP. However, women have a higher incidence of Sphincter Oddi Dysfunction (SOD) [14].

#### 4.1.2. Combined Dormia Basket with ERCP Balloon Dilation

Although gender is an unmodifiable factor, the intervention for choledocholithiasis extraction also implies the presence of modifiable factors that were interesting to evaluate. The introduction of the guide wire through the main route does not lead to a reduction in the risk of PEP in comparison with the injection of contrast substance [17]. Due to this, accessories used during the intervention are more important. In practice, the most common accessories used are extraction probes and dilation balloons. Through repermeabilization, these should help drain pancreatic juices and clear the main bile duct. Manipulation of the pancreatic duct should be avoided as much as possible because the maneuvers will increase the risk of pancreatitis [17].

There are multiple advantages of the biliary stent when treating choledocholithiasis, including the fact that it eliminates stones and maintains the biliary tract’s permeability [18], but this does not necessarily mean that it will also help prevent pancreatitis after ERCP. When an intervention occurs at the Oddi Sphincter or along the biliary or pancreatic pathway, the surrounding organs undergo mechanical, thermal and chemical trauma that results in papillary edema [19]. This papillary edema is also likely to affect the surrounding tissues, blocking normal pancreatic fluid outflow and leading to the appearance of post-interventional pancreatitis. This could be attributed to the fact that using the Dormia probe and the balloon dilation technique may cause a considerable widening of both the papilla and the channels that flow into it, preventing the blockage of pancreatic fluid drainage, even in the event of edema. The simplest solution would have been to place a pancreatic stent, but unfortunately, it has already been proven that they do not effectively prevent pancreatitis [19]. Furthermore, studies show no real benefit even in severe acute necrotizing pancreatitis [20].

A study by DiSario et al. discourages the use of balloon dilation [21] due to the fact that it increases the chance of mortality by PEP. Conversely, another study shows that balloon dilation is safe in Europe and Japan, showing the geographical distribution of the technique’s safety. Therefore, our results regarding using a combined Dormia basket with balloon dilation studies might need validation from centers nearby [22].

### 4.2. Predictive Potential of Biological Markers

One of the key elements of our research is that we assessed the predictive role using both absolute values and ratios to get a more dynamic view of the biological changes that occur in the cases of patients who undergo ERCP. Probably the most useful approach is through blood tests because they provide a quick and accurate response to the patient’s progress. To date, little progress has been made in predicting the occurrence of PEP based on post-ERCP analyses, with even fewer studies that predict the risk of complications among patients.

#### 4.2.1. Pancreatic Enzymes

Statistically significant values were found by far for amylase and lipase, as expected. There is no question that value itself is not extremely important, but rather how much their value increases compared to the normal [23,24]. Acute pancreatitis can be predicted by amylases measured at 4 and 8 h after the intervention [25], but also at 24 h, according to our study (Table 2).

#### 4.2.2. Dynamics of Inflammatory and Infectious Markers

Pathophysiologically, C-reactive protein and neutrophils are not only elevated when an infection occurs in the body but can also rise in pathologies with trauma and inflammation associated [26,27]. From this perspective, the results are very easily corroborated by the fact that choledochal gallstones extraction causes inflammation and trauma locally. Besides the mechanical component of the trauma, there are also chemical, electrical (through electrocauterization) [28], hydrostatic trauma through contrast medium [29] and even allergic lesions [30]. Furthermore, in a study by Arendt et al., one of three main preconditions for the bilio-pancreatic reflux to induce pancreatitis was bacterial superinfection of the biliary secretion, while sterile biliary-pancreatic reflux could not induce pancreatic inflammatory lesions [31]. Therefore, we take into account the infectious element. Furthermore, multilinear regression showed that CRPR is the only variable that can predict higher amylase levels, post-ERCP supporting the necessity for bacterial superinfection added to the traumatic events.

Sang Hoon Lee et al. also managed to identify a ratio model that can forecast the intensity of PEP, but using neutrophils and lymphocytes. This model is worth mentioning due to the special importance it brings to understanding the complexity of the pathology [32]. Even more, the importance of neutrophils and lymphocytes in acute pancreatic pathology was proven a few years before by researchers such as Wang Y. [33] and Jeon T.J [34].

The neutrophil-lymphocyte ratio has become more popular since the COVID-19 pandemic, where numerous studies have proved association with mortality and severity of the disease. It is a direct result of an inflammatory response that causes an increase in neutrophils and a decrease in lymphocyte count [35,36]. However, we need to mention that we did not assess other variable that interferes with NLR, such as hematological malignancies or the use of corticoids [37].

#### 4.2.3. Total Bilirubin Levels on Admission—Paramount Biological Marker in PEP Risk Assessment

Another key outcome of this paper was the notable significance of total bilirubin. Despite being a routine test, total bilirubin above 2.65 mg/mL on admission is a protective factor, indicating a lower risk for PEP. Other clinical multicenter studies showed a similar trend, meaning that normal values are correlated with PEP risk [14]. One study showed that small CBD (Common biliary duct) is associated with the absence of jaundice and a higher risk of PEP [38]. Additionally, Sherman et al. suggested that small CBD is associated with SOD [39]. Even though recent studies proved that patients with small CBD who underwent ERCP are not at risk for PEP, most have normal or lower bilirubin levels [14]. Also, performing endoscopic sphincterotomy (which in our case was done on all patients to minimize the procedure time) on patients with SOD or small CBD is hazardous. It can lead to multiple complications, including pancreatitis, while on the contrary, if the indication is CBD stones, the incidence of these negative events is reduced [40,41]. One review showed that values higher than 1.8 mg/dL of total bilirubin are highly predictive for choledocholithiasis [42]. Also, larger CBD stones are associated with higher levels of TB [43]. Hence, small stones may be seen in patients with lower bilirubin levels and could be easily missed by ERCP [44]. Therefore, it could be the case that Total bilirubin levels on admission might be a surrogate marker for conditions like SOD or non-dilated CBD. In this case, TB is normal or slightly elevated while in the case of CBD stones, the levels of TB on admission are higher.

Even though most predicting factors for PEP are either clinical or procedural-bound, our analysis shows that a routine test like Total bilirubin on admission is much more likely to predict the risk of PEP than any other laboratory markers both in admission and in dynamic. This is a paramount implication for clinicians who need to carefully evaluate patients with normal or slightly increased bilirubin levels upon admission before taking a therapeutic approach, especially if it involves endoscopic sphincterotomy.

Our study has a few limitations that need to be mentioned: We conducted a single-center study with few patients, so there is still a need for confirmation through multicenter studies. Additional markers like, D-dimers, Procalcitonin and Fibrinogen could have been predictive for PEP and were not completely assessed. We also concentrated more on individual factors and less on procedural factors. It is important to mention that this is the first study in the literature that tried to assess the prognostic role of markers through dynamics of pre- and post-ERCP values.

## 5. Conclusions

This study highlights several important findings regarding post-ERCP pancreatitis (PEP) and its predictors in choledochal lithiasis extraction. Regarding gender, female patients remain an important risk group that should be carefully managed to prevent PEP.

The association of different procedures, such as Dormia basket and balloon dilation, in extracting choledocal billiary stones showed positive results.

Furthermore, our investigation explored the utility of various biological markers in predicting PEP outcomes. These markers provide valuable insights for risk stratification and can aid in identifying patients who may be at higher risk of developing PEP.

Interestingly, total bilirubin levels upon admission surpass dynamic markers in their ability to predict PEP outcomes. These results underscore the importance of considering total bilirubin on admission as a primary criterion for patient selection and further investigation to avoid PEP.

By incorporating these insights into clinical decision-making, healthcare professionals can optimize patient outcomes and reduce the incidence of post-ERCP pancreatitis.

## Figures and Tables

**Table 1:** Demographic and clinical data.

Variable	TOTAL	NON-PEP	PEP	*p*-VALUE
Demographic characteristics		86 (64.1%)	48 (35.8%)	
Age (IQR)	67 (57–73)	67 (61–73)	65.5 (47–76.75)	0.492
Sex				0.006
Male	66 (49.2%)	50 (75.8%)	16 (24.2%)	
Female	68 (50.7%)	36 (52.9%)	32 (47.1%)	
----------------------				
History of Cholecystectomy ---------------------- Comorbidities	70 (52.2%)	44 (51.2%)	26 (54.2%)	0.739
Diabetes	22 (16.4%)	14 (16.3%)	8 (16.7%)	0.954
Hypertension	84 (62.6%)	56 (65.1%)	28 (58.3%)	0.436
Atrial fibrillation	21 (15.6%)	13 (15.1%)	8 (16.7%)	0.813
ERCP procedure type				
DB combined with ERCPBD	86 (64.2%)	62 (72.1%)	24 (50%)	0.011
DB combined with BSP	88 (65.7%)	58 (67.4%)	24 (50%)	0.563
ERCP BD combined with BSP	80 (59.7%)	56 (65.1%)	24 (50%)	0.087
All three procedures	68 (50.7%)	48 (55.8%)	20 (41.7%)	0.116
Multiple ERCP’s	26 (19.4%)	20 (23.3%)	6 (12.5%)	0.271

All the variables are expressed as count (%) and compared using a chi-square test. Age is presented as median (IQR) and compared using the Mann-Whitney U test. *p* < 0.05; PEP, Post-ERCP pancreatitis; IQR, interquartile range; ERCP, Endoscopic retrograde cholangiopancreatography; DB-Dormia Basket; ERCPBD, Endoscopic retrograde cholangiopancreatography balloon dilation; BSP, biliary stent placement.

**Table 2:** Laboratory assessment.

Laboratory Findings		On Admission			After ERCP		
	Normal Range	Non-PEP	PEP	*p*-Value	Non-PEP	PEP	*p*-Value
Hematological markers							
Hemoglobin g/dL	12–15	13,1 (12.3–13.9)	13.5 (11.9–14.4)	0.745	12.5 (11.5–13.3)	12.9 (11.4–14)	0.146
WBC × 10^9^/L	4–10	8.1 (6.6–10.6)	7.2 (6.5–10.5)	0.441	8.0 (6.7–10.2)	8.6 (7.7–10.9)	0.073
Neut. Count × 10^9^/L	2–7.5	5.6 (4.1–8.9)	4.7 (3.6–8.5)	0.492	5.2 (4.2–6.3)	6.4 (5.2–8.6)	0.003
NLR		3.1 (1.8–7.3)	3.1 (2.1–6.6)	0.824	3.1 (2.4–4.5)	4.1 (3.0–6.1)	0.006
Tr. Count × 10^9^/L	150–400	250 (196–327)	271 (232–322)	0.419	247 (200–329)	250 (217–309)	0.726
Blood biochemistry							
Amylase U/L	25–125	61 (44–166)	48 (42–76)	0.334	94 (79–138)	881 (363–1443)	<0.001
Lipase U/L	8–78	72 (28–211)	24 (13–103)	0.001	41 (34–64)	1717 (363–2648)	<0.001
AST U/L	9–39	64 (61–121)	82 (49–85)	0.841	69 (48–78)	56 (37–64)	0.553
ALT U/L	3–43	190 (100–213)	80 (63–124)	0.884	169 (76–188)	69 (41–84)	0.985
Creatinine mg/dL	0.7–1.3	0.75 (0.72–0.85)	0.78 (0.61–0.83)	0.114	0.72 (0.71–0.86)	0.75 (0.57–0.92)	0.355
TB mg/dL	0.2–1.2	5.7 (5.4–9.3)	2.8 (1.9–3.8)	0.003	3.6 (2.9–4.8)	2.3 (1.4–4.8)	0.027
CB mg/dL	0–0.5	3.1 (1.4–5.0)	1.1 (0.9–1.9)	0.010	2.7(2.0–3.7)	1.3 (1.0–3.3)	0.006
UB mg/dL	0–0.7	3.9 (2.1–5.0)	1.6 (0.8–3.0)	0.280	0.9 (0.8–1.2)	1.0 (0.4–1.6)	0.248
Total Cholesterol (mg/dL)	109–200	195 (144–239)	188(162–232)	0.478			
Triglycerides (mg/dL)	40–150	145 (87–210)	121 (72–171)	0.103			
Acute phase reactants							
CRP mg/L	0–5	90 (28–160)	25 (10–46)	0.083	42 (35–85)	54 (35–90)	0.626
Fibrinogen mg/dL	170–420	550 (488–620)	530 (323–573)	0.662	510 (481–555)	574 (417–681)	0.710
Coagulation markers							
INR	0.86–1.1	1.1 (1.0–1.3)	1.0 (0.9–1.1)	0.339	1.1 (1.0–1.2)	1.0 (0.9–1.1)	0.705

Parameters were reported as median (IQR) and analyzed using the Mann-Whitney U test. *p* value > 0.05. WBC, white blood cells; Neut. Neutrophils; Tr. Thrombocytes; AST—aspartate transaminase; ALT—alanine transaminase; TB, total bilirubin; CB—conjugated bilirubin; UB—unconjugated bilirubin; CRP, C-reactive protein; INR, international normalized ratio.

**Table 3:** AUC of the proposed variables.

*Variable*	*AUC*	*p-Value*	*CI 95%*	
			Lower Bound	Upper Bound
*CRPR*	0.677	0.001	0.574	0.786
*NLR after ERCP*	0.646	0.006	0.548	0.744
*TB on admission*	0.654	0.003	0.560	0.748
*TBIR*	0.623	0.019	0.521	0.728

*CRPR*—C reactive protein ratio (*CRP* after *ERCP*/*CRP* on admission); *TBIR*—Total bilirubin inverted ratio, TB-total bilirubin, *CRP*—C reactive protein, *NLR*—Neutrophil-Lymphocyte Ratio, *ERCP*—endoscopic retrograde cholangiopancreatography.

**Table 4:** Logistic regression analysis of the proposed markers.

	Univariate Analysis				Multivariate Analysis			
			C.I. 95% for O.R				C.I. 95% for O.R	
	*p*-Value	Odd Ratio	Lower	Upper	*p* Value	Odds Ratio	Lower	Upper
CRPR	<0.001	4.451	2.023	9.789	<0.001	4.337	1.945	9.668
TBIR NLR post-ERCP	0.003 0.004	3.455 3.047	1.525 1.440	7.825 6.448	0.002 0.003	4.004 3.281	1.664 1.490	9.634 7.221
TB on admission	<0.001	4.773	2.002	11.380	<0.001	5.262	2.111	13.113
Gender female	0.009	2.890	1.307	6.398	0.005	2.893	1.377	6.105
Age					0.134	0.977	0.947	1.007
Combined DB with ERCP BD	0.012	2.583	1.237	5.395	0.009	2.890	1.307	6.393
History of Cholecystectomy					0.426	1.382	0.623	3.065
Hypertension					0.704	0.836	0.333	2.100

CRPR—C reactive protein ratio (CRP after ERCP/CRP on admission); TBIR—Total bilirubin inverted ratio, TB—total bilirubin, CRP—C reactive protein, NLR—Neutrophil-Lymphocyte Ratio, ERCP—endoscopic retrograde cholangiopancreatography, DB—Dormia basket, BD—balloon dilation.

**Table 5:** Kruskall-Wallis Test was conducted for each level of Pancreatic enzymes.

Variable	Normal Level of Pancreatic Enzymes	Elevated Pancreatic Enzymes	Post-ERCP Pancreatitis	Kruskal-Wallis Test (*p*-Value)
TB on admission (mg/dL)	2.1 (0.8–4.3)	2.5 (1.0–6.4)	1.5 (0.8–2.3)	0.079
NLR post-ERCP	3.0 (2.4–4.1) *	4.0 (2.9–5.6)	3.9 (3.0–5.9) *	0.014
TBIR	0.8 (0.6–1.2)	0.8 (0.5–1.2)	0.9 (0.6–1.3)	0.342
CRPR	1.0 (0.7–2.4)	0.8 (0.6–1.9) *	1.4 (0.9–6.2) *	0.013

CRPR—C reactive protein ratio (CRP after ERCP/CRP on admission); TBIR—Total bilirubin inverted ratio, TB-total bilirubin, CRP—C reactive protein, NLR—Neutrophil-Lymphocyte Ratio, ERCP—endoscopic retrograde cholangiopancreatography; * flags pairwise significance.

**Table 6:** AUC for predicted probabilities of each multivariable model.

Variable	Area	*p*-Value	Confidence Interval 95%	
			Lower	Upper
TB on admission	0.73	<0.001	0.632	0.829
TBIR	0.698	0.001	0.595	0.798
CRPR	0.710	<0.001	0.612	0.810
NLRpost-ERCP	0.690	0.001	0.588	0.793

## Data Availability

The datasets generated and analyzed during the current study are not publicly available due to institutional restrictions but are available from the corresponding author upon reasonable request.
